# “We all think boots are meant for men”: A community-based participatory assessment of rural women’s barriers to preventing podoconiosis in Rwanda

**DOI:** 10.1371/journal.pgph.0002773

**Published:** 2024-05-03

**Authors:** Gloria Igihozo, Leila Dusabe, Jeanne Uwizeyimana, Esperance Nyiransabimana, Tonya Huston, Janna M. Schurer

**Affiliations:** 1 Bill and Joyce Cummings’ Institute of Global Health, University of Global Health Equity, Butaro, Rwanda; 2 Center for One Health, University of Global Health Equity, Butaro, Rwanda; 3 Heart and Sole Africa, Musanze, Rwanda; 4 Department of Infectious Disease and Global Health, Cummings School of Veterinary Medicine at Tufts University, North Grafton, MA, United States of America; PLOS: Public Library of Science, UNITED STATES

## Abstract

Podoconiosis is a debilitating neglected tropical disease (NTD) that is possibly caused by prolonged exposure to irritant alkaline clay soil. It is endemic to East Africa and disproportionately affects rural female farmers. The condition can be prevented through foot hygiene and regular wearing of protective shoes. In Rwanda, there is limited information on the factors impacting rural female farmers’ access to and utilization of boots while farming. Therefore, this community-based participatory study was conducted to explore the cultural, economic, and ergonomic factors affecting rural farmers’ use of protective footwear. Sixteen audio-recorded focus group discussions were conducted with female and male farmers in four villages with the highest podoconiosis prevalence across four provinces of Rwanda. Transcripts were coded inductively using Dedoose (version 9.0.86) and analyzed through thematic content analysis. Participants expressed that wearing shoes protects against diseases and injuries but ability to afford a pair of protective footwear was a major barrier to accessing and wearing them. There were differences in women and men’s shoe-wearing behaviors while farming, largely driven by the fact that women who wear boots face rumors and backlash. Findings highlight barriers hindering effective podoconiosis prevention among rural female farmers in Rwanda. Opportunities exist to strengthen podoconiosis and NTD prevention programs, through the integration of gender into existing community-based interventions and the inclusion of local communities into the co-designing of contextualized interventions.

## Introduction

Podoconiosis, also known as non-filarial elephantiasis, is a debilitating and progressive health condition characterized by severe swelling of the lower limbs and nodular growths. Research suggests that it is caused by long-term exposure to alkaline clay soil particles that penetrate the skin, triggering an inflammatory response that causes bilateral, progressive, and debilitating lymphedema of the lower legs [1]. Major risk factors for podoconiosis include prolonged occupational contact with alkaline red clay soil, limited access to water, poverty, and genetic susceptibility [2]. People with severe podoconiosis often experience social stigma, poor access to health care, and economic hardship due to reduced income and the additional costs of managing their condition [3].

Podoconiosis is endemic across Rwanda, with prevalence levels ranging from 28 to 119 cases per 100,000 people and an overall prevalence of 68.5 cases per 100,000 people [4]. Although men and women are equally susceptible to the disease, women in Rwanda are disproportionately affected, with a prevalence of 89 cases versus 43 cases per 100,000 among women and men, respectively [4, 5]. This gender disparity is not unique to Rwanda and has also been observed elsewhere in sub-Saharan Africa (SSA) [6]. Women in SSA are thought to be at higher risk of podoconiosis due to extended periods of time spent cultivating and due to societal, cultural, and economic barriers that discourage wearing protective footwear [2, 7].

Podoconiosis is preventable, and swelling potentially reversible, through low-cost interventions that promote skin hygiene and consistent use of protective shoes [8]. In Ethiopia, distribution of shoes among young children in high-risk areas significantly reduced the risk of podoconiosis onset [9]. Shoe wearing has the additional benefit of protecting against physical injuries and other diseases endemic to SSA, such as snakebite, soil-transmitted helminthiases, and schistosomiasis [10, 11]. To be protective, shoes must reduce skin-soil contact substantially, covering both the soles and top of feet. In high soil exposure scenarios, such as farming, boots offer the best coverage and protection. However, such footwear can be expensive for smallholder farmers working in rural areas, especially women [7].

Female farmers in SSA face numerous barriers in using protective footwear, including availability of boots designed ergonomically and aesthetically for women, affordability, decision making power over income, and cultural expectations [7, 12]. In Rwanda, there have been no studies to investigate rural female farmers’ experiences in wearing shoes. This community-based participatory research aimed to characterize the economic, cultural, and ergonomic factors affecting female farmers’ use of protective footwear. The researchers worked with women to understand their experiences and challenges and also to characterize their preferences with the future goal of co-developing and testing new protective footwear for women.

## Methodology

### Study setting

Rwanda is a low-income country in SSA. It is densely populated (~499 persons/km^2^) with approximately 13.2 million residents who are mostly engaged in subsistence cash-crop and livestock production [13]. The country is divided into four administrative provinces (Northern, Southern, Eastern, and Western), and the City of Kigali. Administrative districts are organized hierarchically from province to district, sector, cell, and village. In 2023, the average worker earned 966 USD [14], with male workers earning up to 67% more than female workers [15]. A national mapping exercise conducted in 2017 indicated that the districts with the highest prevalence of podoconiosis in each province were as follows: Nyamasheke District (Western Province), Musanze District (Northern Province), Nyagatare District (Eastern Province), and Nyaruguru District (Southern Province) [4].

All households in Rwanda are categorized into Ubudehe categories, which are socioeconomic stratifications based on household wealth [16]. Since 2015, households have been classified into four Ubudehe stratifications. Categories 1 and 2 are in the lowest quartile and consist of impoverished and vulnerable individuals who have little or no means to own a home, work small part-time jobs, and require government support to afford some necessities (food, health insurance, etc.). Category 3 are middle income earners who don’t require government support and Category 4 are individuals in the wealthiest quartile. Ubudehe categories were revised in 2021 and but the new system is yet to be implemented [17]

### Study design & sampling

A qualitative participatory action research using Focus Group Discussions (FGDs) was conducted between April and June 2022, to understand the perspectives of rural farmers on the use of protective footwear. The study was conducted in the four cells with the highest podoconiosis prevalence in each of the four provinces. These four high prevalence cells were identified by accessing the Rwanda Biomedical Center database of cases recorded during the 2017 national mapping exercise [4]. Four FGDs were held at each study site (Table 1). Potential participants were recruited by first asking Community Health Workers (CHWs) to list all residents in their respective villages who fit the inclusion criteria. To be included in the study, participants had to be aged between 18 and 65 years of age, whose primary source of income is crop and/or animal production, who had lived in the study site for at least one year and belonged to Ubudehe Category 1 or 2. Individuals who were affected by medical conditions that prevent them from wearing commercially available footwear, such as individuals with late stage (Stage 4) podoconiosis, and those who suffered from any mental health disorder that could hinder them from participating in the study, were excluded from the sample. Across all sites, 10 individuals per group were randomly selected from the list and invited to participate, as per standard qualitative FGD guidelines [18], through telephone calls and home visits.

**Table 1:** Focus group discussion participant composition at each study site.

FGD #	Gender	Age range (years)	Health status
1	Female	18–30	Healthy
2	Female	31–65	Healthy
3	Female	18–65	Stage 1 podoconiosis^1^
4	Male	18–65	Healthy

^1^Characterized by mild swelling that is reversible overnight or with leg elevation.

### Data collection

A FGD guide with 11 questions was developed based on key factors affecting use of protective footwear in other SSA contexts: availability of footwear, barriers/facilitators to use, decision-making on household resources, gender norms, culture norms. The guide also facilitated participants’ co-development of a desired farming boot according to the following key criteria: design aesthetics, ergonomics, market price, durability, materials, weight, maintenance, and protection from physical injury or weather. The FGD guide was developed in English and translated to Kinyarwanda. The tool was pre-tested with two groups of female and male farmers outside the study area and was adjusted as needed.

All interviews were held in private rooms at the cell offices to ensure that participants felt comfortable sharing their experiences and opinions. FGDs were conducted in Kinyarwanda, the local language, and lasted between 1.5 to 3 hours. Two female Rwandese team members (J.U. & E.N.) led the discussions, one as the lead facilitator and the other as the note-taker. FGDs were audio recorded, with participants’ consent, and field notes were taken, focusing on main ideas discussed as well as non-verbal cues. At the end of each FDG, participants received educational information on the importance of protective footwear in preventing diseases, such as podoconiosis, and physical injury.

### Data analysis

FGD audio recordings were transcribed verbatim and then translated to English. A codebook was developed after open reading of four transcripts. Two researchers (G.I. & L.D.) coded all transcripts independently and then met to compare codes and resolve any discrepancies. Coding was done inductively and iteratively using Dedoose software (Version 9.0.86). The coding and data analysis process was guided by the constant comparative method in qualitative analysis [19]. Themes and representative excerpts were used to present the study findings. The study methodology and results were developed according to the Standards for Reporting Qualitative Research (SRQR) checklist [20].

### Ethics

This research was approved by the Institutional Review Board at the University of Global Health Equity (Ref: UGHE-IRB/2021/055) and the Rwanda Ministry of Health National Health Research Committee (Ref: NHRC/2022/PROT/016). The research team also sought and obtained approval from each of the four district offices. All participants provided informed written consent prior to participation.

## Results

Sixteen FGDs were conducted with 148 farmers across the four study cells. Of these, 111 were women and 63 were men. From the FGD responses, five prominent themes that reflect the experiences with and factors influencing the use of protective footwear were identified:

1. Shoes protect against diseases and injuries, especially while farming
2. Gender differences exist in shoe-wearing while farming
3. Rumors and backlash discourage women from wearing boots while farming
4. Affordability is a major barrier to accessing and wearing footwear
5. Economic development, good governance, and cultural shifts influence shoe wearing

### Shoes protect against diseases and injuries, especially while farming

All participants expressed that shoes are effective in protecting against diseases and physical injuries. These included podoconiosis, intestinal worms, fungal diseases, as well as physical injuries that could be caused by snake bites, broken bottles, thorns, and other objects that people encountered daily. Other benefits of wearing shoes provided were comfort, hygiene, being able to work efficiently, and walking faster.

*“Wearing shoes is important, I experienced it on our second born, the child had no shoes to wear, we were very much in poverty, the baby became sick, feet were swollen, medical treatment could not heal the child. At Nyagatare Hospital they told me to buy shoes so that they could help the baby. So, I bought boots and the child got healed after wearing them for some time.” (Female, 31–65 years, Nyagatare)*

*“Wearing shoes helps us to be clean and it’s a hygiene practice that contributes to making us healthy. Every Rwandan should wear shoes every day.” (Male, 18–65 years, Gisagara)*

Across all FGDs, there was consensus that wearing shoes has no disadvantage. A few participants said that wearing shoes can be uncomfortable for people with injuries or diseases that make it difficult to wear closed shoes for extended periods of time.

*“Those who see wearing shoes as a challenge are those with the swollen feet who can’t find the size that fits them but regardless, shoes are important” (Male, 18–65 years, Rubavu)*

*“There is nothing bad with wearing shoes except for those who have feet nerve problems. When those people wear closed tight shoes and walk long distance, their feet start swelling.” (Female, 31–65 years, Gisagara)*

Participants stressed that it was also important to wear socks with closed shoes, especially when farming. Socks were useful in keeping feet clean, protecting against blisters, introducing a barrier between shoes and soil, thereby, avoiding bad smells and extending the lifespan of shoes. Although all participants believed that socks held many benefits, a few female participants referred to socks as something worn by men and referred consistently to the benefits that socks have for men who wear boots while farming.

*“When you have showered, oiled yourself, and put your socks on, this will prevent you from having dust on you. This is important for us since we have an issue with getting water on a regular basis and being able to shower as often as one would want.” (Female living with podoconiosis, 18–65 years, Nyagatare)*

*“It is good to wear socks. If a man is going to farm in boots, he puts them on while wearing pants. This will help in a way that the soil does not enter his shoes while cultivating and will prevent any bad smell.” (Female, 18–30 years, Rubavu)*

Overall, participants mentioned that wearing shoes and socks was beneficial in preventing diseases such as podoconiosis, protecting against injuries such as snake bites, and maintaining clean feet. However, shoes could be uncomfortable for people living with diseases that make it hard to wear them.

### Gender differences exist in shoe-wearing while farming

Male and female participants, within their respective FGDs, mentioned that men had better access to and use of protective footwear while farming. Major reasons for this were (1) differences in income, (2) control over household income, (3) limited availability of farming boots for women at local markets, and (4) prioritizing of men’s needs.

*“We don’t have boots [for women] in the village market. There are regular shoes. We would wish to have boots in the village market so that we can buy them.” (Female, 31–65 years, Gisagara)*

*“When I need shoes, I talk to my wife to see if there are funds for it. When we both need them, I buy mine first as I’m the one who does a lot of work. She would have to wait for her turn afterwards.” (Male, 18–65 years, Rubavu)*

*“I cannot decide to buy shoes without consulting my husband, even when it’s money I have earned money through work. I come and ask him if I can buy shoes from that money. He can approve it or say no, for a certain reason. Mostly, women request men’s approval before making a decision.” (Female, 31–65 years, Musanze)*

In addition to this, all female participants said that family responsibilities also influenced their access to protective footwear while farming. On the other hand, only two male participants felt that this was a barrier impacting their access to boots.

*“There may be a chance of earning 20,000 francs, but no woman can spend such amount of money on a pair of boots. Instead, she can spend the money on the children … that situation happens to many women, which means that the way women think about the family is different from the husbands. A woman decides to buy certain things after basic household needs are satisfied.” (Female, 31–65 years, Nyagatare)*

*“The difference between men and women, is that women care more about family well-being than men. It is for this reason that a woman would need to buy shoes but sacrifice her needs and prioritize other things that are needed at home, until she gets to the point of walking barefoot.” (Female, 31–65 years, Gisagara)*

A third barrier difference between men and women was aesthetic appeal. Within all FGDs with female participants, respondents said that boots don’t look good with women’s kitenge wraps, skirts, and dresses. Most women were reluctant to wear boots because it implied that they had to wear pants, which isn’t a norm in their communities.

*“When a woman wears boots and pants, it is not well perceived. It is said to be a violation of cultural norms.” (Female, 18–30 years, Gisagara)*

*“There are times when we put on boots. If your friend sees you wearing them while also wearing a kitenge, they will tell you that they [boots] are meant for men.” (Female, 18–30 years, Rubavu)*

*“From what I have seen or heard, those who wear boots usually put on trousers and tuck them in. If It’s a skirt, she can just put it up and it usually becomes short. [Women] become reluctant to do that because they might laugh at her.” (Female, 18–30 years, Nyagatare)*

Lastly, poor support by spouses and the community discouraged women from wearing boots. Most women stressed that they are discouraged from wearing boots while farming. Contrary to this, however, men felt they were supportive of women wearing boots in the farm but admitted that it’s unusual to see women wearing boots in their communities.

*“The husband cannot encourage the wife to wear the boots, it cannot happen … He purchases for himself only, even when the woman is also financially able to buy for herself. But here in the Eastern Province, no husband can encourage his wife to wear boots. For example, in our household, my sons purchased boots, but their sisters did not. But all of them go to the field, when it is time to harvest sorghum, they all do it.” (Female, 31–65 years, Nyagatare)*

*“Men discourage wives when they see them cultivating wearing boots. Because, even if I tell my husband that I should clean home wearing boots, he asks, ‘do you want to wear the boots I wear?’ [He] emphasizes that [such action] is desiring to take something I do not deserve, and this discourages me from wearing boots.” (Female, 18–30 years, Gisagara)*

*“I can’t protect myself and leave behind my half. What can happen to her can also happen to me, it’s her pain but also, I am the one to look after her when she is sick. Even though I don’t have them [boots] I can buy them [for her] because women have soft skin.” (Male, 18–65 years, Musanze)*

All in all, female and male participants mentioned that there are differences in access to and wearing of protective footwear while farming. Reasons for these differences include differences decision making and purchasing power between men and women, availability of protective footwear for women, family responsibilities, as well as family and social support.

### Rumors and backlash discourage women from wearing boots while farming

All participants said that women who wear boots while farming in their communities often face backlash and rumors. Women who wore boots were perceived as arrogant, trying to show off, trying to become heads of households, or cheating on their husbands. When rural women see other women wearing boots, they mock and laugh at them. Participants also said that although they don’t agree with this practice, there was no way to support women who wear boots because it isn’t the norm.

*“Once, I was in a group of ten women and one came with the boots on. They harassed her, saying that she is arrogant, asking her why she puts boots on while other women do not.” (Male, 18–65 years, Rubavu)*

*“Nothing will be said if they see a man wearing boots while farming, but this won’t be the case if a woman wears them. This is because we all think that boots are meant for men … People might say that such a woman [wearing boots] is full of herself, or that she has a lot of money.” (Female, 18–30 years, Nyagatare)*

*“Boots are great, but [people here] think that women who put on boots are disrespecting their husbands and are probably cheating on them.” (Female, 18–30 years, Musanze)*

Contrary to women’s experiences, male farmers are encouraged to wear boots while farming; those who don’t wear boots are laughed at and those who wear them are deemed intelligent. Some women also admitted that wearing boots is what makes a ‘real man’, so they often associated boot wearing with the male gender, thus making it difficult to accept women who wear them.

*“For men it’s different. When he goes to cultivate without wearing boots, they laugh at him. They question him and wonder why he did not wear boots when he came to cultivate. They face no discouragements [when wearing boots].” (Female, 31–65 years, Musanze)*

*“I do not know how we can call a man that does not have boots. Normally, a real man is man that has boots, one who cultivates wearing boots and who performs other activities wearing boots.” (Female, 31–65 years, Musanze)*

*“When they see a [man] doing all his work wearing boots, they say that the person is intelligent and clean. That he is clean and confident.” (Male, 18–65 years, Gisagara)*

A few participants, on the other hand, mentioned that experiencing such backlash depends on the community and their mindsets. They reflected that there are some communities where it is normal for women to wear boots and added that discouraging each other often comes from the idea that boots are only meant for men. As such, respondents believed that normalizing boot wearing for women within their communities would contribute to more women wearing boots in the farm.

*“It depends to where you come from. Around here, it is prohibited for women to wear boots. So, it’s really an issue with mindsets.” (Male, 18–65 years, Rubavu)*

*“People have different perceptions, some may see [women] wearing boots and say that these people are bragging, that they are showing that they are rich. Maybe in the north where everyone is involved in farming, you find that most of them wear boots. But here many don’t wear them.” (Female living with podoconiosis, 18–65 years, Nyagatare)*

*“The reason to why [women] don’t encourage each other, is because they think boots are meant for men. But if they believed that boots can be worn by everyone, they wouldn’t discourage others or laugh at them” (Female, 18–30 years, Nyagatare)*

Respondents also said that there are some women who don’t face backlash when they wear boots, but this is mostly because of their profession. For instance, female agronomists and women working in factories and mines are expected to wear boots because their jobs require them to do that. Often, when referring to women who wear boots while farming, all respondents said that only female agronomists wear them and admitted that small-scale female farmers are reluctant to wear boots because they don’t want people to think they are pretending to be agronomists.

*“In our community, it is not common to see women wearing boots. Some women who wear boots are teachers- when it is raining heavily. But [the community] often wonders so many things about them. Saying, ‘did she become an agronomist?’” (Male, 18–65 years, Gisagara)*

*“In the past, I believed that everyone who was wearing boots was an agronomist. Even now, people are still confused wondering if [a woman wearing boots while farming] is an agronomist. This makes you wonder when you’ve become agronomist, so you feel discouraged.” (Female, 31–65 years, Nyagatare)*

When asked what can be done to change the status quo, all participants expressed that women and men should encourage each other to wear boots while farming. However, female participants admitted that they need to experience wearing of boots during farming because they can’t encourage other women to practice what they haven’t experienced.

*“The reason husband and wife encourage each other to wear shoes, or a mother reminding child to wear shoes, is that if one is injured, the other will suffer the consequences. If my child is injured or has a problem, I suffer the consequences. It is for that reason that at home, we have to support each other.” (Female, 31–65 years, Musanze)*

*“You would not be able to tell someone about how good something is when you haven’t experienced it. Wearing boots is good, but none of us have ever worn them.” (Female, 18–30 years, Nyagatare)*

Overall, participants expressed that women who wear boots while farming are scorned by the society. Although this depends on one’s community, mindsets, and culture, all participants mentioned that changing the boots wearing behaviors of female farmers requires that women encourage and support each other.

### Affordability is a major barrier to accessing and wearing footwear

Despite the importance of protective footwear, most participants farmed barefoot because of being unable to afford shoes. Those who owned shoes removed them when farming to avoid wearing them out because they did not have the financial means to buy another pair. Even though farmers earn money through selling their harvests, factors such as small yields and droughts affected their financial stability. Consequently, other financial responsibilities such as paying school expenses, feeding the family, and taking care of household needs took priority over buying shoes.

*“Because of my limited financial capability, I walk to the farm in closed plastic shoes but when I get to the land, I remove them and cut grass barefoot.” (Female living with podoconiosis, 18–65 years, Musanze)*

*“The challenge we often face, especially me, is lack of financial means. Because of limited land to cultivate, I earn a small harvest. This short income must address a lot of needs such as meeting the children school requirements, including the school fees or school materials, or school feeding contributions. I also need to feed the household with the limited income. So, those become priorities over buying shoes.” (Male, 18–65 years, Gisagara)*

Participants felt that shoes sold on the local market were too expensive. Moreover, price fluctuations made it difficult to save money for shoes. Some participants, especially those living with podoconiosis, said that shoes available on the local market did not fit them. Most participants owned one pair of good shoes, which were only worn for special occasions such as going to church, weddings, and social functions, among others.

*“Sometimes you can go to the market, and they would tell you that shoes cost 4,000 francs. When you decide to go back, you find that the price has changed, it is higher. When you ask why, they tell you that it’s because of taxes. This can be frustrating for us, as we had budgeted for the price that they had initially told us.” (Female, 18–30 years, Musanze)*

*“Most of the time I wear open plastic shoes [when farming] because the other shoes that I have are to be worn for a trip, work, or going to an important place such as going to church or to visit people.” (Male, 18–65 years, Gisagara)*

Respondents stated that wearing boots was uncomfortable during dry seasons and that it made walking harder when performing household chores such as fetching water. Most participants admitted that the type of shoes worn depended on the activities they were performing, and not necessarily because the shoes were deemed to be protective. Lastly, a few participants mentioned that the decision not to wear protective shoes while farming stemmed from fear that it would damage crops.

*“I try to wear closed plastic shoes during the rainy season and light shoes such as sandals during the dry season. Wearing closed shoes in the dry season will make your feet hot.” (Female, 18–30 years, Rubavu)*

*“When cultivating and weeding, one will need to go in the land barefoot so that they don’t damage the crops. We wear [shoes] only when we are walking from home or back home. (Male, 18–65 years, Musanze)*

All in all, ability to access and wear protective footwear depended on participants’ financial means, the availability of good quality shoes on the market, the cost of shoes, the weather, and the types of farming activities being done. Although most participants admitted to owning at least one pair of shoes, they stated that they choose to farm barefoot to preserve the shoes that they own.

### Economic development, good governance, and cultural shifts influence shoe wearing

Participants agreed that Rwandese culture and views around shoes has changed over time. In the past, shoes were worn only by rich people. With time, this tradition changed, and people have embraced wearing shoes. These shifts were attributed to the country’s development; namely, the introduction of government policies and initiatives meant to encourage shoe-wearing.

*“[When I was a child], I went to school wearing shoes bought for me by my uncle. When I got to school the teachers beat me and asked why I was wearing shoes. I left the shoes at school and went back home; my parents took me back to school and apologized to the teacher for letting me wear the shoes. From that day, I never went to school wearing the shoes … hygiene practices were not cared for. Then, compared to today, things have changed. When my children are going to school, I have to check if they have showered and see that their shoes are clean.” (Female, 31–65 years, Nyagatare)*

*“It has become a culture to wear shoes. The Government of Rwanda is encouraging us to wear shoes. It has even become compulsory to enter the market wearing shoes.” (Male, 18–65 years, Gisagara)*

Although the people’s perspective around shoe wearing has changed overtime, participants mentioned that the culture and mindset of the past still influences some people’s decision to wear shoes, especially amongst older populations. However, respondents said that people who hold onto these practices believe that there are no harms associated with being barefoot.

*“I think many people who don’t wear shoes, it’s because of traditional culture. In the past people were not wearing shoes, so they dwell in such traditional practices and don’t want to stop walking barefoot.” (Male, 18–65 years, Gisagara)*

Despite government policies and laws to encourage shoe-wearing, some individuals only wear shoes when going to places where they would be seen by authorities but remain barefoot when no one is watching. Other respondents stressed that wearing shoes while farming has not become a cultural practice. Therefore, they wear shoes in public places but farm barefoot. Lastly, even though shoe wearing has become common, wearing boots while farming is still seen as a practice of the rich.

*“Wearing shoes makes you look good. However, there are still people who choose to walk barefoot while at home and only wear shoes when going to church.” (Female, 18–30 years, Nyagatare)*

*“Some of the people believe that wearing boots while cultivating is for rich people. So, it’s the reason they do not wear them [when farming].” (Female, 18–30 years, Gisagara)*

Overall, the culture of wearing shoes has changed over time, with the implementation of different government laws and regulations. Although the shoe-wearing culture is now common, some people still hold on to practices of the past and majority of the Rwandan society still views wearing shoes while farming as an uncommon practice that is for the rich.

### Women’s perspective on ideal farming footwear (boot)

Women described the characteristics of ideal farming boots in Table 2.

**Table 2:** Desired farming footwear characteristics developed by female participants.

Characteristic	Female farmers’ preference
Height	Medium- proportionate to height, lighter than tall boot, still protective
Material	Plastic- lightweight, waterproof, easy to clean, good quality
Weight	Lightweight- especially for muddy season, walking long distances
Soles	Medium thickness to protect against sharp objects, provide traction
Fasteners	None- boots should be waterproof ventilation at top preferred
Aesthetic	Black to hide dirt or blue/green with logo (to differentiate from men)
Cleaning	Easy to clean with brush or soap/water, quick dry, no smell

## Discussion

In this assessment of rural female and male participants understood the importance of footwear in avoiding injury. However, affordability, gender-based differences in household decision-making, male-focused aesthetics of locally available boots, community ridicule and rumors against female boot-wearers reduced their use. Protective footwear is a key pillar of global and national strategies to reduce the burden of NTDs such as podoconiosis, leprosy, soil transmitted helminthiases, and snakebite [21, 22]. Moreover, programs aiming to improve occupational health and safety of workers in key employment sectors, such as agriculture, mining, construction, and manufacturing, require strong adherence to personal protective equipment use [23]. Our study, highlighting key gender differences in access and use of protective equipment, demonstrates the value of gender-responsive approaches in driving social and behavioral change. Such improvements are critical to achieving Government of Rwanda Vision 2050 goals, and especially, those related to economic development, improved standards of living, gender equity, and improved health [24, 25].

Major barriers to access and use of farming boots in Rwanda included affordability, availability of boots at local markets, and competing household priorities. This is similar to other SSA contexts, such as Ethiopia, where limited financial resources and poor availability of shoes at village markets hindered their use [7, 12]. Efforts to mitigate these barriers have included donating shoes, subsidizing the cost of shoes for rural communities, and implementing interventions where local communities can be trained to manufacture shoes [8, 26, 27]. For instance, the Mossy Foot Treatment and Prevention Association in Ethiopia established workshops where podoconiosis patients were trained to manufacture footwear. This improved patients’ access to shoes and lowered the cost of the footwear [26]. Although these interventions have so far been reported in Ethiopia only, they show promising impact and are worth adapting to other resource-limited contexts. Therefore, given Rwanda’s ongoing efforts to develop and strengthen local value chains and domestic markets through the ‘Made in Rwanda’ policy [28], such initiatives have the potential to boost local production and lower the cost of protective footwear for rural Rwandese.

Across the four study districts, rural women who wore boots were ridiculed and rumored to be cheating on their spouses. These findings align with those from rural northern Ethiopia where women rarely wore shoes popular among men, also for fear of ridicule and gossip [7]. In both studies, backlash not only came from the wider community, but was also perpetuated by other women. Mitigating backlash that is tied to cultural norms and stereotypes requires collective social change and transformation [29]. Because gender intertwines with axes of culture and power to determine access to NTD programs, it’s necessary for NTD programs to conduct gender-sensitive analyses of how different communities perceive, experience, and interact with disease risk, prevention, and treatment to develop targeted and contextualized interventions [30, 31]. The World Health Organization’s 2030 NTD roadmap encourages nations to implement gender-equity focused NTD programs, through community-based education focusing on behavior change and addressing stigma and discrimination impacting effective prevention and treatment [32]. Therefore, it is important for NTD programs to implement community-wide advocacy campaigns targeting peer intimidation and dispelling gender-based cultural stereotypes and norms affecting rural women’s utilization of protective footwear. Rwanda’s NTD strategic plan outlines the country’s efforts to conduct community sensitization campaigns on shoe-wearing [22]. Integrating a gender component into existing education campaigns, aiming to address gender norms surrounding boots-wearing, would help the country make further progress towards its goal to eliminate podoconiosis as a public health threat.

The research uncovered that female participants needed their husbands’ permission to spend household and personal income. Men, however, reported purchasing items independently or, sometimes, sharing such decisions with their spouses. This disparity in control over income and personal agency is a challenge faced by rural female farmers across SSA [33, 34]. Most male heads of household control all family income and assets, leaving women with little to no income for personal expenses [34–37]. These dynamics limit their ability to make decisions that benefit their wellbeing, such as buying boots, and have been documented in several NTD programs including mass drug administration (MDA) campaigns [38, 39]. The intersection of decision-making and gender norms on women’s autonomy to participate in NTD programming contributes to delays in access to information, diagnosis, and treatment initiation and threatens the effectiveness of existing programs [39, 40]. NTD programs across the globe are tasked with implementing interventions that empower women to make choices about their health and well-being, whether it be through co-designing interventions that are sensitive to their needs or involving them in leading and running community-based programs [41–43]. Our study findings reiterate this call and highlight the need for increased participation of women in NTD program design, implementation, and leadership to address disparities in access to NTD prevention and treatment services and ensure that women aren’t left behind. Additionally, because men play a pivotal role in health-related decision-making and ultimately influence the success of NTD programs [38, 44], gender-sensitive interventions should involve men in dismantling gender stereotypes that hinder women from actively participating in prevention and treatment efforts to create a supportive environment where women are safe to make decisions about their health. Moreover, the misaligned perceptions between men and women regarding household decision-making suggest there is a need to address women’s financial autonomy in any gender sensitive programming.

This study should be interpreted in light of a few limitations. The sampling frame did not include people suffering from severe forms of podoconiosis, whose experiences and barriers might differ from the general population. To mitigate this, study participants included individuals suffering from stage 1 (mild) podoconiosis and those at high risk of developing the condition. Discussions were conducted in groups, which might have led some participants to express opinions that are socially favorable. During FGDs, researchers reminded participants that it’s acceptable to have differing opinions and encouraged them to respect each other’s perspectives. Lastly, results of this study are specific to rural female farmers in Rwanda and might not be generalizable to other contexts. Despite these limitations, the study sheds light on previously undocumented social, cultural, economic, and ergonomic barriers hindering Rwandan rural female farmers from accessing and wearing protective footwear and highlights important policy and programmatic intervention gaps that need to be addressed for effective podoconiosis eradication.

## Conclusion

In this community-based participatory assessment, Rwandan female and male farmers shared important financial and gender-based barriers to shoe-wearing, despite understanding the value of protecting their feet. Such challenges impede government efforts to reduce the burden of podoconiosis, as well as other NTDs and occupational injuries, and ultimately impact efforts to combat poverty. Specific interventions, such as integrating gender into community-based health programming, addressing the stigma faced by women who wear boots, investing in local shoe manufacturing, and supporting women to design and produce footwear that is appealing to rural female farmers could address barriers to accessing and using protective footwear.

## Supporting information

S1 File English focus group discussion guide. (DOCX)
