# Supplementary material for: “We all think boots are meant for men”: A community-based participatory assessment of rural women’s barriers to preventing podoconiosis in Rwanda
Source: PLOS Glob Public Health. 2024 May 3;4(5):e0002773. doi: 10.1371/journal.pgph.0002773 (PMC11068164; doi:10.1371/journal.pgph.0002773)
Supplement: S1 File — (DOCX) [file pgph.0002773.s001.docx]

# **English Focus Group Discussion Guide**

Moderator:

Notetaker:

District:

Sector:

Date:

Time started/ended:

Number of participants:

Participant group:

*[Facilitator script]*

Hello, my name is [________] and I am a researcher from the University of Global Health Equity. This is my colleague [__________].

The purpose of this project is to understand the factors that influence shoe wearing, especially during farming. We are here to learn about your experiences and to hear your ideas and opinions. This information will help us understand the challenges and opportunities in shoe wearing among rural female farmers and will help the research team design a preferred footwear based on your experiences and recommendations.

***[Ask participants to read through the consent form and answer their questions. Explain that participants should not share information that is sensitive or private in a group setting.]***

If you consent to participating in this discussion, may I please ask you to sign the form?

***[Participants and facilitators sign consent forms.]***

Thank you for agreeing to participate. Before we begin, I want to remind you that there are no right or wrong answers. We welcome a diverse array of opinions, perspectives, and experiences. Please do not hesitate to share any information that you may deem as different or contradictory to the information shared by other participants. There is no compensation for participating in this study but we will provide transportation support.

To help us have a fruitful discussion, please listen to the following ground rules:

- Only one person speaks at a time
- There is no order on who speaks first, anyone who has something to share is welcome to start speaking
- Kindly avoid interrupting each other
- Please respect participants who are sharing opinions that differ from your own

Does anyone have any questions they would like to ask before we begin?

***[Assign participants a number from 1 to 10]***

Okay, I will turn on the recorder and we can begin our conversation.

***[TURN ON THE RECORDER]***

1. **Ice breaker activity**

Let’s go around the circle and introduce ourselves. State your first ID number, your age, how long you have been a farmer, the type of farming you do, and what you like most about your community.

***[As participants are speaking, please fill in the following table]***

| ID | Age | Sex | Years farming | Type of farming (livestock, crops, both) |
| --- | --- | --- | --- | --- |
| *E.g. 9* | *23* | *F* | *2* | *Crops* |
|  |  |  |  |  |
|  |  |  |  |  |
|  |  |  |  |  |
|  |  |  |  |  |
|  |  |  |  |  |
|  |  |  |  |  |
|  |  |  |  |  |

***[Use the following questions to guide the discussion. Allow time for participants to respond to each other’s comments and encourage all participants to speak. Ask follow up questions when an answer is unclear.]***

1. While visiting your community, we see that some people wear shoes while others go barefoot. What is the reason for this?

1. Are there advantages to wearing shoes? Are there disadvantages?
2. What are the advantages or disadvantages to walking barefoot?
3. Is wearing shoes related to health? Are there diseases you can prevent by wearing shoes?
4. Are there cultural rules around wearing shoes or walking barefoot?
5. Are there differences between children and adults?
6. Is it important to wear socks?

2. Let’s think about the times when people wear shoes or go barefoot.

*[Facilitator guides participants to fill in this chart. Check marks can be put in multiple boxes when participants disagree.]*

| **Activity** | None | Open plastic | Open leather | Closed plastic | Closed leather | Closed canvas | Boots |
| --- | --- | --- | --- | --- | --- | --- | --- |
| Household chores (cooking, cleaning the house or yard, washing clothes) |  |  |  |  |  |  |  |
| Cultivating crops |  |  |  |  |  |  |  |
| Cutting grass |  |  |  |  |  |  |  |
| Grazing livestock |  |  |  |  |  |  |  |
| Cleaning livestock pen |  |  |  |  |  |  |  |
| Fetching water |  |  |  |  |  |  |  |
| Collecting firewood |  |  |  |  |  |  |  |
| Market |  |  |  |  |  |  |  |
| Social activities (e.g., church) |  |  |  |  |  |  |  |

3. We have heard from you when people wear shoes and what types they wear. What factors influence whether a person wears shoes while farming?

a. Is there a shoe type that is ideal for cultivating or rearing livestock?

b. Does weather or season play a role?

*[Probe: comfort, aesthetic design, hygiene, keeping shoes clean, traction on soil, cost, social expectations]*

4. What are the best shoes to wear while farming?

a. What do they look like?

b. What are the best shoes for grazing livestock or cleaning their pens? What are the best shoes for the other cultivation crops?

*[Probe: other outdoor activities - cutting grass, fetching water, collecting firewood]*

5. Where are good shoes made? Are good shoes made in Rwanda?

a. Where do you buy your shoes?

b. Are the shoes you want available nearby?

c. Are there problems getting the shoes you want? What are they?

d. What is a reasonable amount to pay for shoes used for farming? What can people in your community afford to pay?

6. Have you noticed any differences in shoe wearing between men and women in your community? If so, could you share with us your experience?

1. Who goes barefoot more often? What do people say about men or women who wear shoes while farming?
2. Are there differences between men and women in purchasing shoes?
3. When women in your community wish to buy shoes, do they need to ask their husbands for permission? Do men need to ask their wives for permission?
4. Who makes decisions about spending money in the household, men or women? Or both?
5. How could men and women encourage each other to wear shoes while farming or working outdoors?

7. Do women encourage or discourage each other to wear boots while farming?

1. What do women say if they see another woman wearing boots?

8. Do men encourage or discourage women to wear boots while farming?

1. What do men say?

9. What influences your style?

a. Social media

b. Friends, family

c. People from big cities such as Kigali

10. Let’s imagine that we could design the perfect footwear for farming. How would they look?

*[Facilitators should prepare the flip chart with some known Target Product Plan features but add to the criteria list based on previous discussion points]*

| **TPP Criterion** | **Features** |
| --- | --- |
| Height (short, tall) |  |
| Material (plastic, leather, canvas) |  |
| Weight |  |
| Sole |  |
| Fasteners |  |
| Durability/lifespan |  |
| Waterproof vs breathable |  |
| Aesthetic design |  |
| Colours |  |
| Cleaning |  |
| *[Other…]* |  |

Are there any features that I have left out?

11. Do you have any other points to add about footwear or gender?

*[Conclude the discussion]*

Thank you for receiving us warmly and for taking time to share your experiences and opinions with us. Your responses were thoughtful, interesting, and invaluable to this study. Before we conclude, we would like to share some information about why shoes are important to our health.

#

# **Shoe illustrations to accompany FGDs (English)**

***[The images have been removed due to copyright considerations.]***

| **Type of shoes** | **Illustrations** |
| --- | --- |
| **Boots** |  |
| **Closed leather** |  |
| **Closed plastic/foam** |  |
| **Open plastic** |  |
| **Open leather/Leather Sandals** |  |
| **Closed canvas:** |  |

# 
